# Evaluation of the Efficacy and Safety Profile of Long-Pulsed 1064 Neodymium:Yttrium-Aluminum-Garnet (Nd:YAG) Laser in Hemangioma and Vascular Malformation in Darker Skin Types

**DOI:** 10.7759/cureus.25742

**Published:** 2022-06-08

**Authors:** Manmit Kaur Hora, Nishant Choudhary, Surbhi Agrawal, Shreya Gupta, Jagriti Gandhi, Abhishek De, Gobinda Chatterjee

**Affiliations:** 1 Dermatology, The Derma Clinic, Ludhiana, IND; 2 Department of Dermatology, Ram Krishna Dharmarth Foundation (RKDF) Medical College Hospital and Research Center, Bhopal, IND; 3 Department of Dermatology, Lakshmi Narain (LN) Medical College and Research Center, Bhopal, IND; 4 Department of Dermatology, Calcutta National Medical College and Hospital, Kolkata, IND; 5 Department of Dermatology, Institute of Postgraduate Medical Education and Research, Kolkata, IND

**Keywords:** lasers for hemangioma, hemangioma, safety study, hemangioma treatment, port-wine stain, dark skin, laser in dermatology, lasers in vascular malformation, nd:yag laser

## Abstract

Introduction

A prospective, interventional study was conducted to evaluate the efficacy and safety profile of long-pulsed neodymium:yttrium-aluminum-garnet (Nd:YAG) laser in the treatment of vascular lesions in the darker skin patients of Fitzpatrick skin type IV and V.

Materials and method

The study was conducted at a tertiary care hospital. Institutional ethical committee permission was obtained before starting the study. Twenty-nine patients presenting with vascular lesions were enrolled in the study. The patients were called once a month for sessions for six months. Clinician Global Impression (CGI) scores were used for evaluation. We followed a “per protocol” analysis.

Results

Of the 29 patients we enrolled, three dropped out for various logistic reasons, and 26 patients completed their treatment. After six months of follow-up of the 26 patients who completed their treatment, 12 (46.15%) had shown complete healing (CGI = 4, 70%-100% improvement in lesions). The rest of the 14 (53.84%) patients showed good improvement (CG1 = 3, reduction of 50%-70% of lesions). No permanent side effects were noted.

Conclusion

Long-pulsed 1064 Nd:YAG laser proves to be an effective treatment for hemangioma and vascular malformation in darker skin patients with its major advantages of being a safe, well-tolerated, cost-effective procedure with minimal downtime and minimal side effects.

## Introduction

Vascular lesions such as hemangioma and port-wine stain (PWS) are often a cause of concern for the patients. They cause a huge physical and psychological impact on the patients, especially the females. Medical treatments are often found to be unsatisfactory and accompanied by various side effects. Treatment is often sought for reasons such as psychological stress due to cosmetic disfigurement, pain, ulceration, and bleeding. In the late 1960s and early 1970s, argon lasers were used to treat vascular lesions. Although these lasers were effective, they were not considered a first-line therapy because they often caused side effects such as scarring and permanent dyspigmentation. The use of lasers increased after Anderson and Parrish described the theory of selective photothermolysis [1]. Side effects reduced significantly, as now physicians were using laser after determining appropriate parameters such as wavelength, fluence, and pulse duration to selectively target hemoglobin within blood vessels without damaging the surrounding tissue.

Pulsed dye laser (PDL) is considered to be the gold standard of treatment for vascular malformations and hemangioma. However, PDL has limitations in the Indian context because of the high price of the machine, high maintenance cost, limited efficacy, and risk of epidermal damage in darker skin types.

Long-pulsed neodymium:yttrium-aluminum-garnet (Nd:YAG) laser has been found to be effective in vascular lesions in studies conducted by Coles et al. [2] and Levy et al. [3]. However, there are very few studies of similar nature on darker skin types. We undertook a prospective, interventional study to know the efficacy and safety profile of long-pulsed Nd:YAG laser in the treatment of vascular lesions in the darker skin patients of Fitzpatrick skin type IV and V.

## Materials and methods

The study was conducted at a tertiary care hospital. Institutional ethical committee permission was obtained before starting the study. Dermatology outpatient department patients presenting with hemangioma and vascular malformation and having isolated cutaneous slow-flow vascular malformation who fulfilled the inclusion and exclusion criteria were included in the study. Twenty-nine patients were enrolled in the study. The patients were subjected to laser treatment after proper evaluation. The patients were counseled about the possible effects and side effects of the procedure in their vernacular language. Written informed consent was taken from every patient. In the case of minors, informed consent was taken from their parents/guardians.

Patients who were unable or unwilling to give informed consent and with unrealistic expectations were excluded from the study. Pregnant and lactating females and patients with connective tissue disorders, psoriasis, vitiligo, keloidal tendencies, active local infection, psychiatric illness, and high flow malformation (arteriovenous malformation) were also excluded from the study. Long-pulsed 1064 Nd:YAG laser was used for treatment. The patients had to undergo six sessions. Each session was placed one month apart. Once included, the patient was evaluated by a Doppler study of the lesion to assess the depth, extent of the lesion, and possible association of arteriovenous malformation. Other investigations such as complete blood count, bleeding time, clotting time, and biopsy were done if deemed necessary. Test spots were done before commencing treatment.

Pulse duration, fluence, and pulse count were decided and adjusted according to the type of the lesion, patient’s skin type, area of involvement, and patient’s reaction to the therapy. After the procedure, topical antibiotics and sunscreen were applied over the treated area. The patients were sent home the same day with directions to apply topical antibiotics (if required) for 2-3 days and avoid sun exposure for at least a week, along with continued use of sunscreens. The results were recorded according to the Clinical Global Impression (CGI) as per the investigator on the basis of the photography. For each patient, at every session, details of laser parameters used, CGI score, effects, and side effects were recorded in case record forms.

Statistical analysis

Clinician Global Impression (CGI) scores were used for evaluation. We followed a “per protocol” analysis. Observations at baseline and after the sixth session were studied for statistical analysis to assess efficacy. Only those patients who completed the entire study period (baseline and follow-up sessions) were considered for final analysis. Mann-Whitney U-test was used to calculate the p-value.

## Results

A total of 29 patients were included in the study, with 62% (n = 18) female and 38% (n = 11) male. The age of our patients ranged from six months to 45 years. The patients belonged to Fitzpatrick skin type IV and V. Most (n = 20) patients belonged to Fitzpatrick skin type IV. Twelve (41.3%) patients had port-wine stains, and 17 (58.6%) patients had hemangioma. Twenty-two (75.85%) had lesions from birth, whereas seven (24%) patients developed them later in life. Of the seven patients who had developed lesions after birth, the majority (n = 5, 17.2%) had developed lesions by the age of one month. However, two of our patients developed lesions after infancy; one was diagnosed as adult-onset port-wine stain, and the other was diagnosed as verrucous hemangioma. Among 17 cases of hemangioma, 11 had their lesions started from birth, whereas among PWS, all except one had their lesions starting from birth. Most (n = 19, 64.5%) patients had their lesions localized to the head and neck. Other involved sites were the upper limb (n = 6, 20.6%), trunk (n = 3, 10.3%), and lower limb (n = 2, 6.8%).

Of the 29 patients we enrolled, three dropped out for various logistic reasons, and 26 patients completed their treatment. Of the 26 patients who completed their treatment, 12 (46.15%) had shown complete healing. The results were evaluated using Clinician Global Impression scores.

Hemangioma showed a negative correlation between age of presentation and improvement (measured by CGI score at the final follow-up visit at six months) stating that, with the increase in the age of presentation, chances of complete recovery decrease. Such a correlation was not shown by PWS (Table 1).

**Table 1:** Correlation between age of presentation and CGI score at six months Correlation between age of presentation and CGI score at six months by Spearman’s rank correlation

	Hemangioma (n = 17)	Port-wine stain (n = 9)	Total (n = 26)
Correlation coefficient (rho)	-0.05	0	-0.26
95% confidence interval	-0.52 to 0.44	0	-0.59 to 0.14

**Figure 1:**
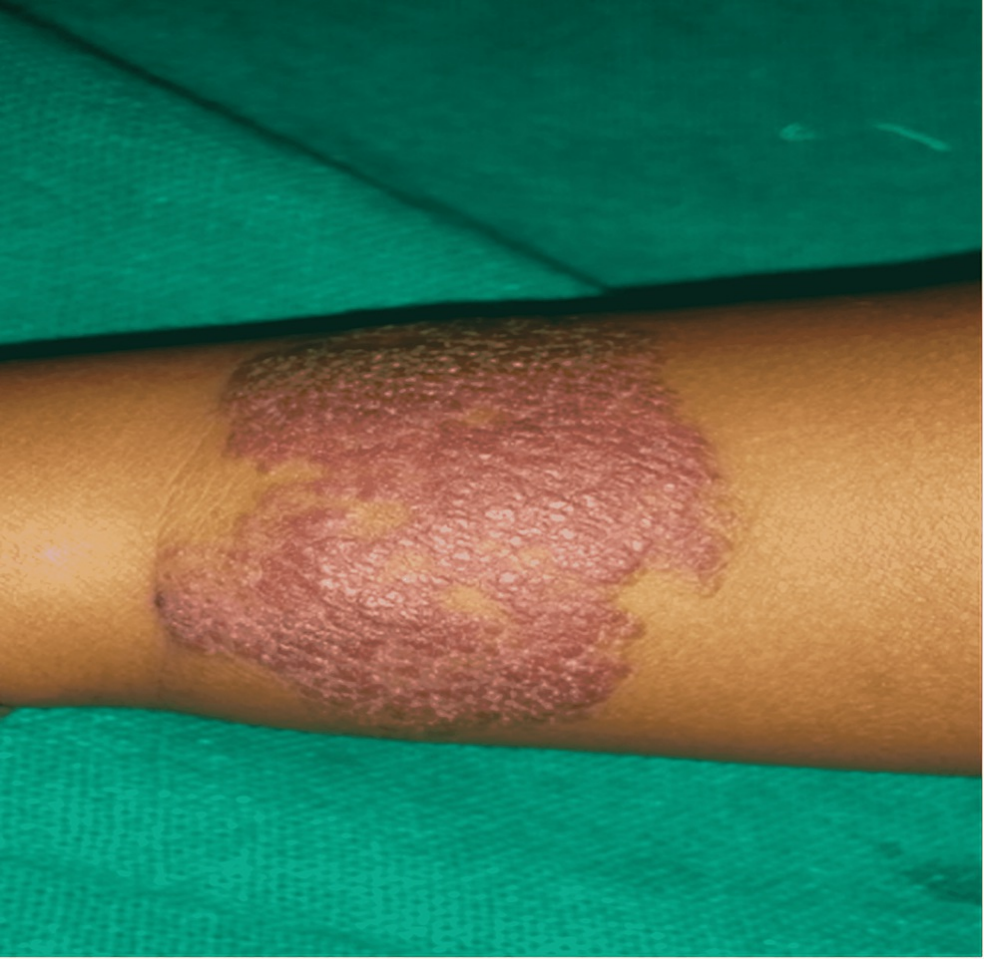
Clinical image of a two-year-old child with a hemangioma on the upper limb

**Figure 2:**
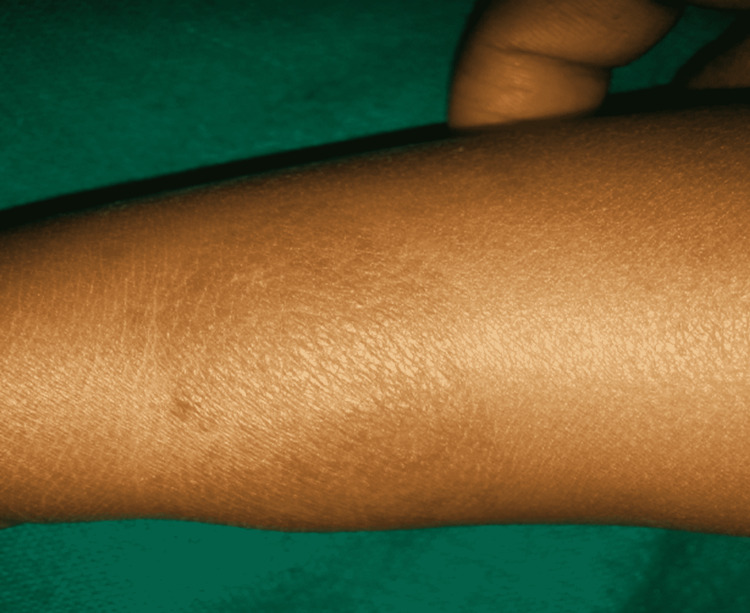
Improvement noted after six sessions of long-pulsed 1064 Nd:YAG laser

The changes in the CGI score over six months are shown in Table 2. Scores were comparable at baseline and decreased from baseline significantly (p < 0.001) in both the treatment arms. Also, when individual follow-ups were compared to baseline, they varied significantly (p < 0.001) in both three and six months in the hemangioma group. Figure 1 shows the clinical image of a two-year-old child with a superficial hemangioma on his upper limb. Figure 2 shows the clinical image of the same child after six sessions of long-pulsed 1064 Nd:YAG laser.

**Table 2:** Changes in the CGI score over six months p-value for between-group comparisons using Mann-Whitney U-test *p < 0.001 for within-group comparison between the baseline visit and the particular visit (Freidman’s ANOVA followed by post hoc Dunn’s test)

Visits	Hemangioma (n = 17)	Port-wine stain (n = 9)	p-value (between groups)
Baseline (mean ± SD, median (IQR))	0.47 ± 0.52, 0 (0, 1)	0.22 ± 0.44, 0 (0, 1)	0.306
Three months (mean ± SD, median (IQR))	2.59 ± 0.62*, 3 (2, 3)	1.89 ± 0.60, 2 (1.5, 2)	0.029
Six months (mean ± SD, median (IQR))	3.65 ± 0.49*, 4 (3, 4)	3 ± 0*, 3 (3, 3)	0.008
p-value (within groups)	<0.001	<0.001	

**Figure 3:**
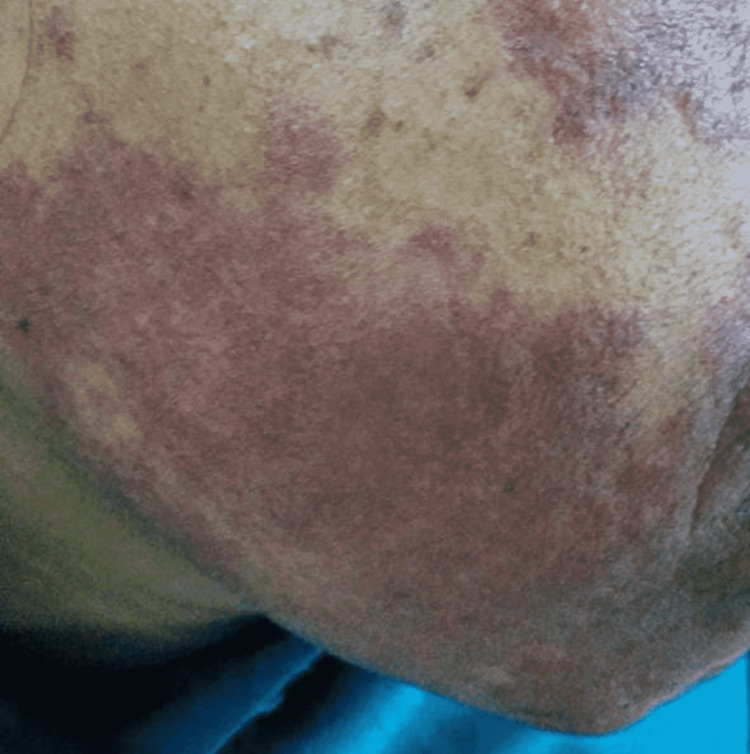
Port-wine stain at the beginning of treatment

**Figure 4:**
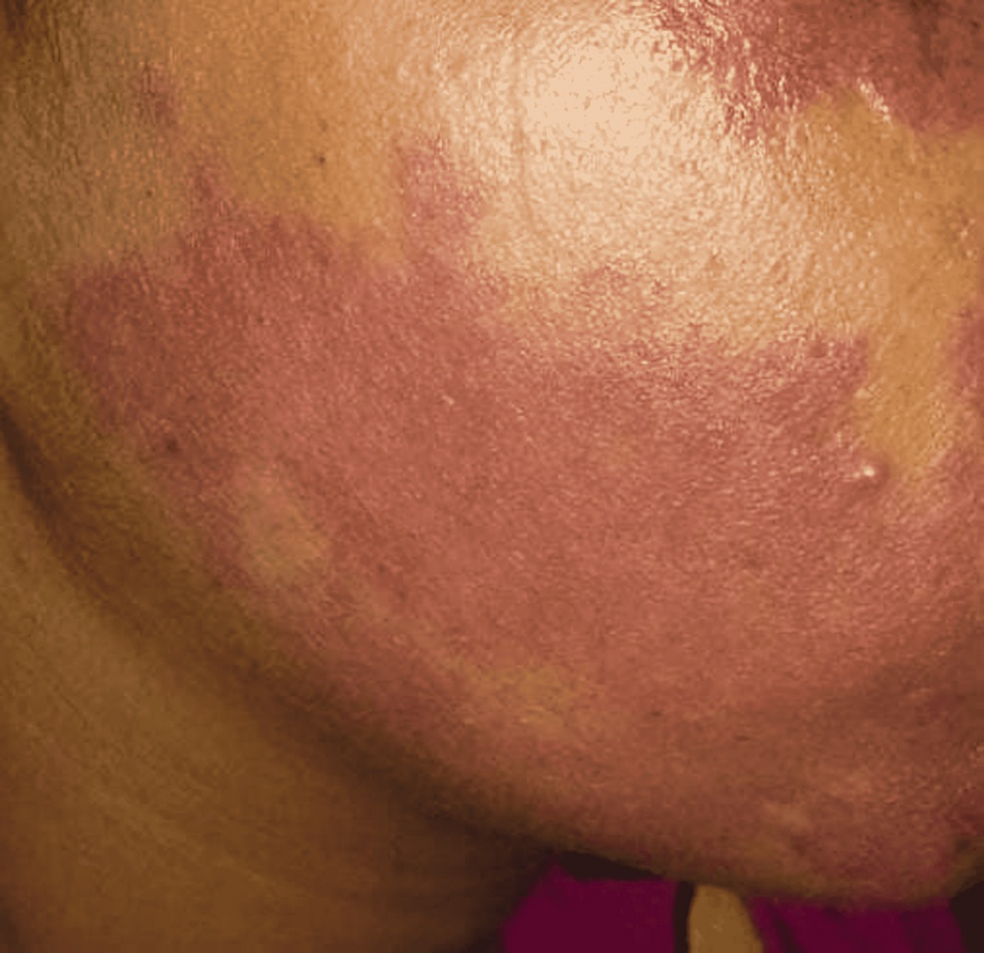
Port-wine stain after six sessions of long-pulsed 1064 Nd:YAG laser

In the port-wine stain group, only the final follow-up varied significantly from baseline. The CGI score in the port-wine stain group was significantly less (Table 2) than that of the hemangioma group post-baseline in all ensuing follow-ups. Figure 3 shows the clinical image of an adult with a port-wine stain on the right side of the face at the beginning of the treatment. Figure 4 shows the clinical image of the same adult after six sessions of long-pulsed 1064 Nd:YAG laser.

No permanent side effects were noted in our study. Two patients developed postoperative bleeding, which stopped after putting continuous pressure for five minutes. The common side effects noticed in our study were erythema, pain, and burning sensation. Erythema along with burning sensation was seen in 15 patients. Pain was seen in 12 patients. These side effects subsided in a day or two.

## Discussion

Vascular malformation and hemangioma are one of the most common tumors in childhood. Most hemangiomas do not require treatment as they undergo spontaneous resolution by the age of one year. In a prospective, randomized controlled trial on 121 infants, Batta et al. found that laser treatment in uncomplicated hemangiomas is no better than a wait-and-see policy as nearly 42% of children improved without any treatment [4]. Treatment is often sought for cosmetic reasons or for some complications that interfere with day-to-day functioning. Hemangiomas can be treated by oral beta-blockers, topical beta-blockers, surgical procedures, corticosteroids, cryotherapy, and lasers [5].

Lasers have been used to treat vascular lesions since the 1960s [6]. There have been many reports of the treatment of these lesions using different types of lasers such as argon laser, carbon dioxide laser, Nd:YAG laser, potassium titanyl phosphate (KTP), pulsed dye laser (PDL), long-pulsed tunable dye laser, and intense pulsed light (IPL) [7-13]. Before selecting a laser or IPL for the treatment of skin lesions in patients of darker skin types, one should be cautious as darker skin types are more prone to develop side effects [14].

PDL is considered the gold standard treatment for port-wine stains and hemangioma. Satisfactory results have also been seen with argon laser, but because of the risk of scarring, its use is limited [15]. PDL is likely to cause adverse effects such as hypopigmentation and skin atrophy [4]. This limits the use of the PDL laser in children and patients with Fitzpatrick skin type IV and V.

Landthaler et al. observed that the result of PDL in superficial hemangiomas was good but was poor in the case of deeper parts of hemangiomas [16]. They, however, concluded that the Nd:YAG laser is the treatment of choice for thick hemangiomas because of its deeper penetration (8 mm) in the tissue and less scattering of the beam [16]. We used the Nd:YAG laser because of its deeper penetration and its safety profile in darker skin.

The number of female patients (n = 18, 62%) was higher compared to males (n = 9, 38%) in our study, which corroborates with data of a larger study done on 110 patients by Vlachakis et al. (female, 74.5%; male, 25.5%) [17]. The higher number of female patients could be attributed to the fact that they constitute a group of our population that are more affected cosmetically by these afflictions and hence have a higher tendency to seek treatment.

The most common site of hemangioma and vascular malformation in our study was the head and neck region (65.5%), followed by the upper limb (20.6%), trunk (10.3%), and lower limb (6.8%), which is similar to that seen in the study by Clymer et al. [18].

PDL has been considered to be a gold standard among lasers for the treatment of vascular tumors and malformation, and there is extensive literature on its use; however, only a few studies could be found where long-pulsed Nd:YAG laser has been used for treating hemangioma. To date, very few studies have been done on type IV and V skin to treat vascular tumors with long-pulsed 1064 Nd:YAG laser. Twenty-six patients in our study completed their treatment. The patients were of Fitzpatrick skin type IV and V.

The treatment outcome in our study was measured using Clinician Global Impression (CGI) scores at baseline and follow-up at three and six sessions. At six-month follow-up of the 26 patients who completed their treatment, 12 (46.15%) had shown complete healing (CGI = 4, 70%-100% improvement in lesions). The remaining 14 (53.84%) patients showed good improvement (CG1 = 3, reduction of 50%-70% of lesions). A similar study was conducted by Vlachakis et al. in Greece. In that study, 30 out of 38 patients had completed treatment. They found that six months after the completion of the third session, 24 (80%) patients had shown complete resolution, and six (20%) had shown good improvement (50%-89% reduction in the size of the lesions) [17].

However, their study differed from ours in the following aspects. They have used a technique of applying ice for 10-15 minutes preoperatively and causing vasoconstriction in the lesions before the application of the laser beam. They used a power of 35-45 W and a pulse length of 2-10 ms. The patients were given general anesthesia and admitted postoperatively for a few days until the postoperative edema had settled. The average hospital stay was 1-2 days [17].

In our study, the treatment was given as a daycare procedure, without any sedation. No pre-procedure cooling was done; however, post-procedure cooling was done by applying ice for five minutes. The patients were discharged on the same day under the cover of appropriate topical antibiotics and sun protection. The parameters used in our study were a power of 40-50 W given with a pulse duration of 8-10 ms in hemangioma and a pulse duration of 5-8 ms for PWS.

Superficially, the results of Vlachakis et al. may look superior [17]; however if we take the improvement in sheer numbers, we would like to highlight the fact that ours was an outpatient procedure, requiring no sedation or hospital stay, thus improving better compliance. Also, it makes the treatment relatively cost-effective since we have obviated the incurring cost of general anesthesia and hospital stay.

Although PDL is the gold standard laser for treatment, it is known for complications such as post-inflammatory hyperpigmentation, scarring, keloid formation, and bleeding. In our study, we did not come across any major complications. Immediate complications such as erythema and pain were noticed. Out of the 29 patients, two developed postoperative bleeding, which stopped spontaneously after putting continuous pressure for five minutes. No patient reported atrophic scars in our study. The rate of complications was similar to other studies. Preeyanont et al. performed a long-pulsed Nd:YAG Laser on 160 patients with hemangioma between 1989 and 1993 and noted side effects such as slow healing, superficial skin necrosis, and occurrence of scars in 10% of patients [19]. Vlachakis et al. reported atrophic scars in six (5.8%) patients and hypertrophic scars in two (1.9%) patients, whereas one patient had postoperative bleeding [17]. One of the biggest drawbacks of PDL in the treatment of vascular tumors in darker skin has been the induction of post-inflammatory hyperpigmentation. None of our patients reported pigmentary changes after the completion of therapy.

The Nd:YAG laser has been used for the treatment of vascular tumors for well over two decades now; however, there was a paucity of literature about its efficacy and safety in darker skin types. The results of our study show that long-pulsed Nd:YAG laser is an effective treatment modality for hemangioma even in darker skin patients and has minimal side effects.

PDL has been used for many decades for the treatment of vascular malformations such as PWS; however, very few studies to date have shown the efficacy of long-pulsed Nd:YAG laser in the treatment of PWS. In our study, there was a significant reduction in the size of port-wine stain after six months of treatment compared to baseline. Seven (58%) out of 12 patients with PWS showed a CGI score of 3 (50%-70% reduction). Albeit slow, there is an improvement in PWS.

Most of the studies done to see the efficacy of Nd:YAG laser in vascular tumors have been in children only. In our study, we recruited both adults and children. An interesting fact that came out in our study was that hemangiomas responded better in children than in adults. Hemangioma showed a negative correlation between age of presentation and improvement (measured by CGI score at the final follow-up visit at six months), stating that, with the increase in the age of presentation, chances of complete recovery decrease. Such a correlation was not shown by PWS.

The limitation of our study is that due to the small sample size, the results cannot be generalized. Due to the limitation of resources, a head-to-head comparison of PDL and long-pulsed Nd:YAG laser for vascular lesions could not be performed. Further studies are needed to study the efficacy and safety of laser procedures in darker skin people.

## Conclusions

Long-pulsed 1064 Nd:YAG laser proves to be an effective treatment for hemangioma and vascular malformation even in darker skin patients with its major advantages being a safe, well-tolerated, cost-effective procedure with minimal downtime and minimal side effects. It is an important tool for dermatologists in the management of this rare but challenging problem. However, its limitation remains to be slow improvement in PWS.
